# Gambling among forensic psychiatric patients: A prevalence study

**DOI:** 10.1192/j.eurpsy.2025.1514

**Published:** 2025-08-26

**Authors:** J. W. Eriksen, L. U. Sørensen, S. Kristiansen, M. D. Terkildsen, R. M. Hansen, T. Marcussen, L. Frostholm

**Affiliations:** 1 The Reseach Clinic on Gambling Disorder, Aarhus University Hospital; 2 Department of Forensic Psychiatry, Aarhus University Hospital Psychiatry; 3 Department of Clinical Medicine, Faculty of Health, Aarhus University, Aarhus N; 4 Department of Sociology and Social Work, Aalborg University, Aalborg; 5 DEFACTUM, Central Denmark Region, Aarhus N, Denmark

## Abstract

**Introduction:**

While gambling problems are increasingly recognized as a public health concern, knowledge of the prevalence and impact within psychosocially vulnerable populations is scarce. Never before has the prevalence of gambling problems been studied in a forensic psychiatric patient population. Given the risk for gambling problems to exacerbate existing mental health conditions, cause financial strain, and contribute to criminal behavior, understanding its prevalence and associated factors within this population is critical.

**Objectives:**

The study aims to estimate the prevalence of gambling behavior and gambling problems among forensic psychiatric patients in Denmark and compare the results to the general Danish population. Additionally, the study seeks to identify associations between psychosocial and demographic variables and the occurrence and severity of gambling problems. Lastly, the study will explore gambling motives and their relation to gambling problems in this patient population.

**Methods:**

This observational, cross-sectional study will include inpatients and outpatients in Central Denmark Region forensic psychiatric care and inpatients in Capital Denmark Region forensic psychiatric wards from June 1 to December 31, 2024. Participants must be 18, and speak Danish, English, or Greenlandic. Data will be collected via self-report questionnaires and electronic patient records. Key measures include gambling behavior (past year gambling, frequency, and expenditure), gambling problems (the Problem Gambling Severity Index), and gambling motives (the Gambling Motives Questionnaire-Financial).

**Results:**

Data collection ends by December 31, 2024; preliminary findings will be presented at the conference.

**Conclusions:**

Results will provide valuable insights into the prevalence and characteristics of gambling problems in this population, informing clinical care and potentially guiding the development of targeted interventions. By highlighting the need for attention to gambling issues in forensic psychiatric settings, the study may enhance rehabilitation and recovery efforts and successful discharge by addressing potential risk factors for recidivism.

**Disclosure of Interest:**

None Declared

